# A Pyroptosis-Related Gene Signature for Predicting Survival in Glioblastoma

**DOI:** 10.3389/fonc.2021.697198

**Published:** 2021-08-17

**Authors:** Xin-Yu Li, Lu-Yu Zhang, Xue-Yuan Li, Xi-Tao Yang, Li-Xin Su

**Affiliations:** ^1^Department of Interventional Radiotherapy, Shanghai Ninth People’s Hospital, Shanghai Jiao Tong University School of Medicine, Shanghai, China; ^2^Department of Neurosurgery, Shanghai Ninth People’s Hospital, Shanghai JiaoTong University School of Medicine, Shanghai, China; ^3^Department of Surgery, The First Affiliated Hospital of Zhengzhou University, Zhengzhou, China

**Keywords:** glioblastoma, pyroptosis, overall survival, prognosis, signature

## Abstract

**Background:**

In this study, a prognostic model based on pyroptosis-related genes was established to predict overall survival (OS) in patients with glioblastoma (GBM).

**Methods:**

The gene expression data and clinical information of GBM patients were obtained from The Cancer Genome Atlas (TCGA), and bioinformatics analysis of differentially expressed genes was performed. LASSO Cox regression model was used to construct a three-pyroptosis-related gene signature, and validation was performed using an experimental cohort.

**Results:**

A total of three pyroptosis-related genes (*CASP4*, *CASP9*, and *NOD2*) were used to construct a survival prognostic model, and experimental validation was performed using an experimental cohort. Receiver operating characteristic (ROC) analysis was performed, and the area under the ROC curves (AUC) was 0.921, 0.840, and 0.905 at 1, 3, and 5 years, respectively. Functional analysis revealed that T-cell activation, regulation of T-cell activation, leukocyte cell-cell adhesion, and positive regulation of cell adhesion among other immune-related functions were enriched, and immune-related processes were different between the two risk groups.

**Conclusion:**

In this study, a novel prognostic model based on three pyroptosis-related genes is constructed and used to predict the prognosis of GBM patients. The model can accurately and conveniently predict the 1-, 3-, and 5-year OS of GBM patients.

## Introduction

Glioblastoma multiforme (GBM) is the most common and most aggressive human brain tumor. Increasing incidence of GBM is becoming evident in patients with advanced age (1). It is estimated that more than 15,000 people die each year from GBM in the USA (2). Formulating a treatment plan for GBM patients requires a multidisciplinary treatment approach (3). Despite several innovations in the treatment of GBM, it remains one of the most difficult and complex cancers to treat, and the median survival is approximately 1 year (4). The multifactorial etiology of GBM makes prognostic prediction challenging. Therefore, considering the limited treatment strategies for GBM, novel prognostic models should be developed, to accurately and conveniently predict the overall survival (OS) of GBM patients.

Pyroptosis is a new type of programmed cell death mediated by the gasdermin D protein and characterized by the release of inflammatory mediators (5). Recently, pyroptosis has become a research hotspot in cancer initiation and progression. Besides, it is reported to be closely related to gastric cancer, colorectal cancer, hepatocellular carcinoma, breast cancer, skin cancer, and malignant mesenchymal tumors (6–12). Recent studies on the relationship between pyroptosis and cancer are providing new research ideas for the prevention and treatment of cancer. However, whether pyroptosis-related genes are correlated with the prognosis of patients with GBM remains largely unknown.

In the present study, mRNA expression data and corresponding follow-up clinical information of GBM patients were obtained from the Cancer Genome Atlas (TCGA) database and GTEx web portal. A prognostic multigene signature was then constructed with pyroptosis-related differentially expressed genes (DEGs). Functional enrichment analysis was performed to explore the potential mechanisms associated with the identified DEGs.

## Materials and Methods

### Datasets

The corresponding clinical information and mRNAs-seq data for GBM were downloaded from the TCGA Data Portal (https://portal.gdc.cancer.gov/repository). Normal tissues were matched TCGA adjacent tissue and GTEx normal tissue. A total of 33 pyroptosis-related genes from previous systematic reviews were extracted and are provided in ** Table S1** (13–17). A total of 62 GBM patients were recruited from The First Affiliated Hospital of Zhengzhou University as the validation cohort.

All validation cohort patients gave written informed consent, and ethical permission was obtained from The First Affiliated Hospital of Zhengzhou University.

### Identification of Differentially Expressed Pyroptosis-Related Genes

Normalization of the read count values was performed using edger (R package). To identify the differentially expressed genes (DEGs) between tumor tissues and adjacent normal tissues, the “limma” R package was used with FDR <0.05 and |log2FC| ≥1 in the training cohort.

### Construction of the Prognostic Signature

The prognostic values of DEGs were determined by univariate Cox analysis, and genes significantly related to OS of GBM patients were identified. To avoid the overfitting problem, least absolute shrinkage and selection operator (LASSO)-penalized Cox regression analysis was performed. The penalty parameter (λ) adjustment was performed by tenfold crossvalidation based on minimum criteria (**Figure S1**). The survival genes were selected and used to construct a prognostic model based on the multivariate Cox regression analysis results. Each GBM patient was assigned an individual risk score, and the risk score formula was defined based on the expression level of each gene and the regression coefficient derived from the multivariate Cox regression model. The prognostic signature as risk score = $\Sigma_{i=1}^{n}\exp_{i}*\beta_{i}$ (where n, exp_i_, and β_i_ represent the number of prognostic genes, the expression value, and the coefficient of gene i, respectively). Patients were divided into high-risk and low-risk groups based on the median values of the risk score. Principal Component Analysis (PCA) was carried out in R using the prcomp function from the stats package and visualized with the ggbiplot library. For the survival analysis of each gene, the “surv_cutpoint” function of the “survminer” R package was used to determine the optimal cutoff values of the risk scores. In addition, a time-dependent receiver operating characteristic (ROC) curve analysis was performed using the R package “survivalROC” to evaluate the discrimination ability of the gene signature.

### Validation of the Prognostic Signature by Quantitative Real-Time PCR and Western Blot

To validate the prognostic model, tumor tissue and normal tissue were collected from 62 GBM patients from The First Affiliated Hospital of Zhengzhou University. Using the same risk score formula, the risk score of each patient in the validation cohort was calculated, and patients were classified into the high- or low-risk groups based on the median-based cutoff values. Multivariate Cox analysis revealed that *CASP9* was an independent prognostic factor. Ethical approval was obtained from the First Affiliated Hospital of Zhengzhou University Ethics Committee.

### Quantitative Real-Time PCR

Total RNA was extracted from the target tissue samples and thoroughly ground in a mortar under liquid nitrogen. To lyse the cells, 1 ml of Trizol reagent (Life Technology, Grand Island, NY, USA) was added, and the sample was incubated for 15 min at room temperature on a shaker. To assess the mRNA expression level, the RevertAid First Strand cDNA Synthesis Kit (Thermo Scientific, Lithuania) was used to synthesis the first-strand cDNA. Quantitative PCR was performed using Roche LightCycler^®^ 480 Real-Time PCR System with SYBR^®^ Green qPCR mix 2.0 kit. The primers used in this study were obtained from TsingKe biological technology (Nanjing, China), including *CASP9* (forward 5′-CTGTCTACGGCACAGATGGAT-3′, reverse 5′-GGGACTCGTCTTCAGGGGAA-3′), *β-actin* (Forward: 5′-CGAGCACAGAGCCTCGCCTTTGCC-3′, Reverse: 5′-TGTCGACGACGAGCGCGGCGATAT-3′). The relative mRNA levels were calculated by the 2-ΔΔCt method.

### Western Blot

Western blot was performed to determine the protein expression level. Samples were isolated and lysed in RIPA buffer with protease inhibitors. Proteins (40 μg) were separated using SDS-PAGE with 10% acrylamide gels. Western blot analysis was performed using antibodies against mouse monoclonal antibody-anti-human CASP9 (9502) from Cell Signaling Technology, and mouse monoclonal antibody-anti-human β-actin (sc-47778, Santa Cruz Biotechnology), followed by incubation with horseradish peroxidase (HRP)-coupled mouse secondary antibody (1:10,000, NA93, GE Healthcare). To confirm equal protein loading, the blots were reprobed with a β-actin antibody, and analysis of the data was performed using NIH ImageJ software.

### Functional Enrichment Analysis

Kyoto Encyclopedia of Genes and Genomes (KEGG) and Gene Ontology (GO) enrichment analyses of the DEGs were performed using the “clusterProfiler” R package and the Cytoscape plugin “ClueGO.” The infiltrating score of 16 immune cells and the activity of 13 immune-related pathways were calculated using the single-sample gene set enrichment analysis (ssGSEA) method in the Gene Set Variation Analysis (GSVA) package of R software (18, 19). Besides, correlation analysis between the expression levels of the three pyroptosis-related DEGs and infiltrating immune cells was performed using ssGSEA.

## Results

### Identification of Prognostic Pyroptosis-Related DEGs

**Figure 1:**
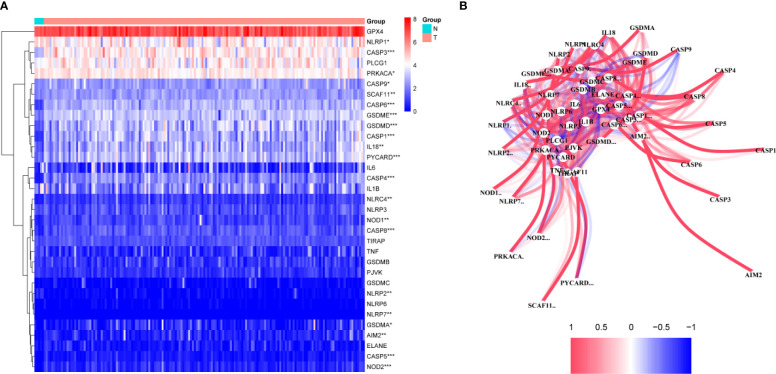
Identification of the candidate genes. **(A)** Heatmap of the differential gene expression in the two groups. **(B)** The relationship between these genes.

**Figure 2:**
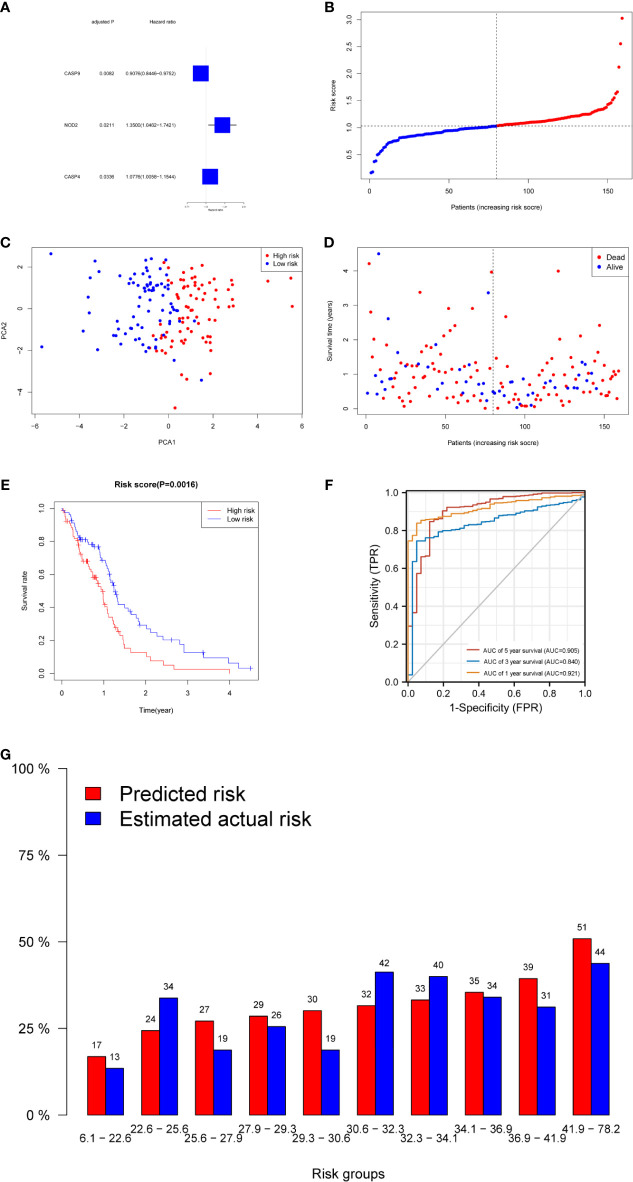
Prognostic analysis of the three-gene signature model in the derivation cohort. **(A)** Forest plots showing the results of the univariate Cox regression analysis between gene expression and OS. **(B)** The distribution and median value of the risk scores in the derivation cohort. **(C)** PCA plot of the derivation cohort. **(D)** The distributions of OS status, OS, and risk score in the derivation cohort. **(E)** Kaplan–Meier curves for the OS of patients in the high-risk group and low-risk group in the derivation cohort. **(F)** AUC of time-dependent ROC curves verified the prognostic performance of the risk score in the derivation cohort. **(G)** Calibration plot for predicted versus estimated actual risk.

A total of 130 normal tissue samples were downloaded from the TCGA and GTEx databases, while 169 GBM tumor tissue samples were obtained from the TCGA database. **Tables 1**, **2** show the clinicopathological data of the GBM patients included in the study. A total of 19 pyroptosis-related genes were found to be differentially expressed between the tumor and adjacent non-tumor tissues. Differential gene expression in the two groups was represented in a heatmap (**Figure 1A**, blue: low expression level; red: high expression level). The relationship between the genes is presented in **Figure 1B**. To build a prognostic model for GBM, the training dataset was used and LASSO Cox regression analysis was performed to identify stable markers from the survival-related genes. To find an optimal *λ*, 10-fold crossvalidation based on the minimum criteria was employed, and the final model gave a minimum crossvalidation error. A total of three DEGs (*CASP4*, *CASP9*, and *NOD2*) were identified. **Figure 2A** illustrates the results of multivariate Cox regression.

**Table 1:** Clinical characteristics of the GBM patients used in the derivation cohort.

Characteristic	Levels	Overall
n		169
Gender [n (%)]	Female	59 (35.1%)
	Male	109 (64.9%)
Race [n (%)]	Asian	5 (3%)
	Black or African American	11 (6.6%)
	White	150 (90.4%)
Age [n (%)]	≤60	87 (51.8%)
	>60	81 (48.2%)
Age [median (IQR)]		60 (50.75, 69)

**Table 2:** Clinical characteristics of the GBM patients used in the validation cohort.

Characteristic	Levels	Overall
n		68
Gender [n (%)]	Female	30 (44.1%)
	Male	38 (55.9%)
Age [n (%)]	≤65	32 (47.2%)
	>65	36 (52.8%)
OS event [n (%)]	Alive	34 (50%)
	Dead	34 (50%)
Age [median (IQR)]		66.5 (56.5, 72)

### Construction and Validation of the Prognostic Model

**Figure 3:**
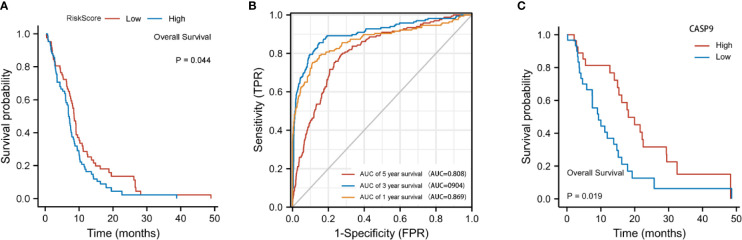
Validation of the three-gene signature in the validation cohort. **(A)** Kaplan–Meier curves for the OS of patients in the high-risk group and low-risk group in the validation cohort. **(B)** AUC of time-dependent ROC curves verified the prognostic performance of the risk score in the validation cohort. **(C)** Patients with a low *CASP9*expression level exhibited a poor prognosis.

The risk score was calculated using the following formula: 0.01204*expression level of *CASP4*+0.22295* expression level of *NOD2*-0.08962*expression level of *CASP9*. A median cutoff value was applied to stratify patients into a high-risk group (*n* = 76) and a low-risk group (*n* = 78) (**Figure 2B**). The PCA showed that patients in the two risk groups were distributed in different directions (**Figure 2C**). As shown in **Figure 2D**, patients in the high-risk score group were closely associated with a high risk of mortality, while patients in the low-risk group had a higher probability of survival. Kaplan–Meier analysis showed that the probability of survival was significantly higher in the low-risk group compared with patients in the high-risk group (**Figure 2E**, *p* < 0.05). The time-dependent ROC curve was carried out to estimate the performance of the risk prediction model. The AUC of the prognostic risk assessment model for the three pyroptosis-related genes was 0.921, 0.840, and 0.905 at 1, 3, and 5 years, respectively (**Figure 2F**). The calibration curve was close to the ideal curve, indicating that the model had a good prognostic effect (**Figure 2G**). In the validation cohort, the risk score of each patient was calculated using the same formula and cutoff value. The overall survival of patients in the high-risk group was significantly worse compared with the low-risk group (*p* < 0.05) (**Figure 3A**). Besides, the AUC of the three-gene signature was 0.869, 0.904, and 0.808 at 1, 3, and, 5 years, respectively (**Figure 3B**). In the validation group, the association between *CASP9* and patient outcome was analyzed. The KM survival curve showed that low *CASP9* gene expression patients had a significantly worse prognosis (**Figure 3C**).

### Expression Level of *CASP9* in GBM Tissues

**Figure 4:**
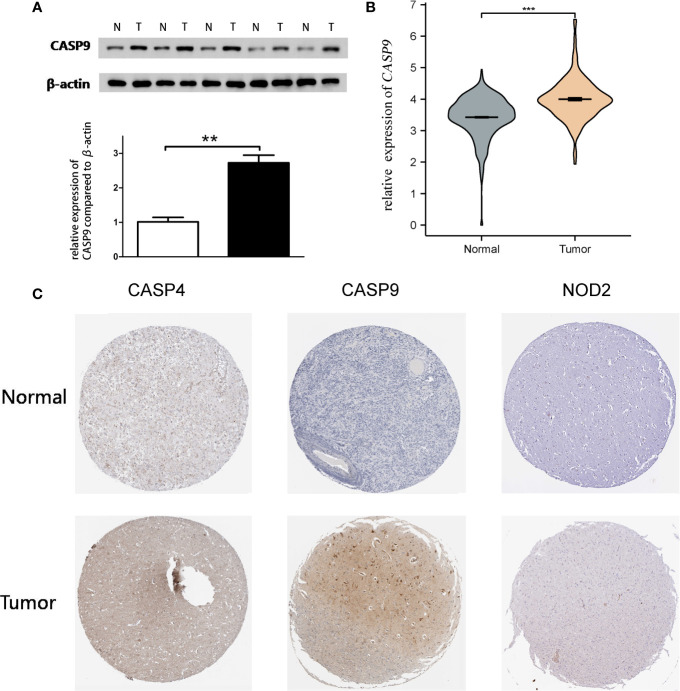
Quantitative real-time PCR (qRT-PCR) and Western blotting. **(A)** Western blot analysis showed a clear overexpression in protein expression levels of *CASP9* in GBM. **(B)** Results of qRT-PCR analysis. **(C)** The level of three pyroptosis-related DEGs (*CASP4*, *CASP9*, and *NOD2*) protein in GBM tissue was upregulated compared with normal tissue. **P < 0.01; ***P < 0.001.

Quantitative real-time PCR (qRT-PCR) and Western blot analysis were performed to detect *CASP9* expression. The results showed that *CASP9* expression in GBM was significantly higher than in adjacent tissues (**Figures 4A, B**). Besides, protein expression of the three pyroptosis-related DEGs was upregulated in GBM tissues compared with normal tissues (**Figure 4C**).

### Independent Prognostic Value of the Risk Model

**Figure 5:**
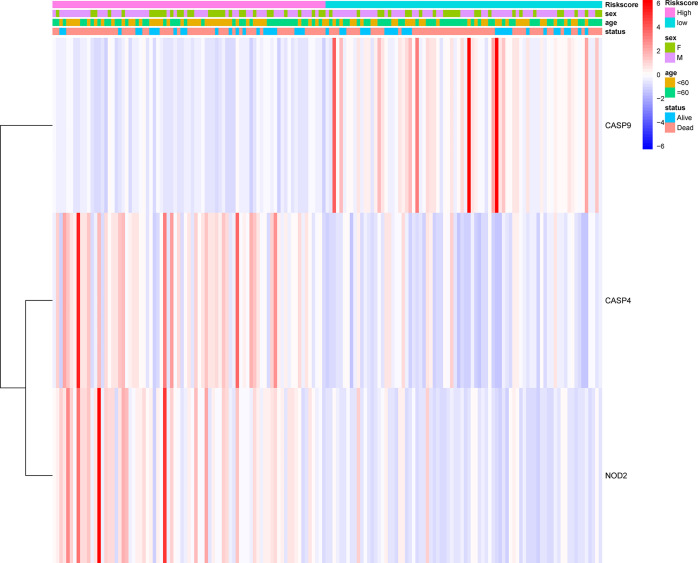
Heatmap (blue: low expression; red: high expression) for the connections between clinicopathological features and the risk groups.

The prognostic significance of different clinical features in patients with GBM was assessed. Univariate Cox regression analysis was used to identify the univariate predictors, and the risk score was found to have a prognostic significance in OS (HR = 2.392; 95% CI, 1.861–3.075; **Table 3**). Even after adjusting for confounding factors, the risk score was found to be a prognostic factor (HR = 1.726; 95% CI, 1.302–2.287; **Table 3**) in GBM patients. The three-gene signature was experimentally validated as an independent prognostic factor (**Table 4**). **Figure 5** shows a heatmap of the pyroptosis-related DEGs and different distributions of patients’ age and survival status between the low- and high-risk subgroups.

**Table 3:** Univariate Cox and multivariate Cox regression analyses in derivation cohort.

Characteristics	Univariate analysis		Multivariate analysis	
	Hazard ratio (95% CI)	*p*-Value	Hazard ratio (95% CI)	*p*-Value
Gender (male *vs*. female)	1.262 (0.988–1.610)	0.062	–	–
Age (>60 *vs*. ≤60)	4.668 (3.598–6.056)	<0.001	3.916 (3.002–5.109)	<0.001
Race (Black or African American *vs*. Asian)	1.470 (0.414–5.214)	0.551	–	–
Risk score (high *vs*. low)	2.392 (1.861–3.075)	<0.001	1.726 (1.302–2.287)	<0.001

**Table 4:** Univariate Cox and multivariate Cox regression analyses in validation cohort.

Characteristics	Univariate analysis		Multivariate analysis	
	Hazard ratio (95% CI)	*p*-Value	Hazard ratio (95% CI)	*p*-Value
Gender (male *vs*. female)	1.026 (0.719–1.466)	0.887	–	–
Age (>60 *vs*. ≤60)	1.365 (1.073–1.415)	0.07	1.358 (0.967–1.905)	0.077
Risk score (high *vs*. low)	1.419 (1.009–1.997)	0.044	1.412 (1.004–1.986)	0.048

### DEG-Based Tumor Classification

**Figure 6:**
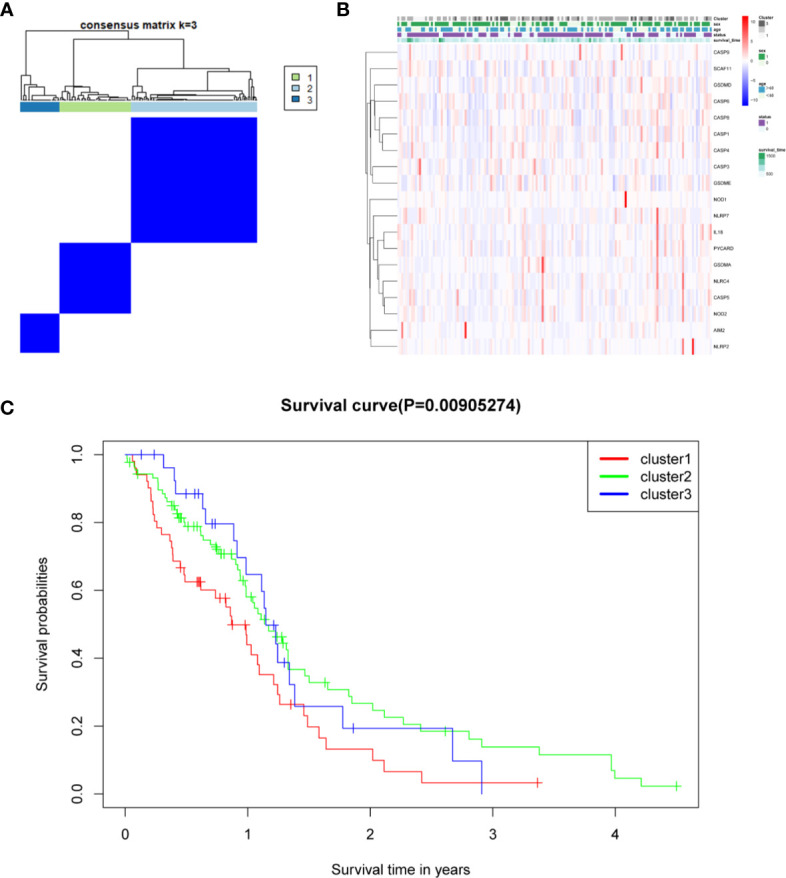
Tumor classification based on the pyroptosis-related DEGs. **(A)** One hundred sixty-nine GBM patients were grouped into three clusters according to the consensus clustering matrix (*k* = 3). **(B)** Heatmap and the clinicopathological characters of the three clusters classified by these DEGs. **(C)** C Kaplan–Meier OS curves for the three clusters.

The relationship between the expression of pyroptosis-related DEGs and GBM subtypes was explored. Consensus clustering analysis was used to classify GBM patients into subtypes. By increasing the clustering variable (*k*) from 2 to 10, the highest intragroup correlation and low intergroup correlation were observed when *k* = 3 (**Figure 6A**). The results showed that the 169 GBM patients were divided into three clusters based on the 19 DEGs. The gene expression profile was matched to the clinical data and presented in a heatmap (**Figure 6B**). The OS in the three clusters was compared, and significant differences were reported (**Figure 6C**).

### Functional Analyses and Infiltrating Immune Cells

**Figure 7:**
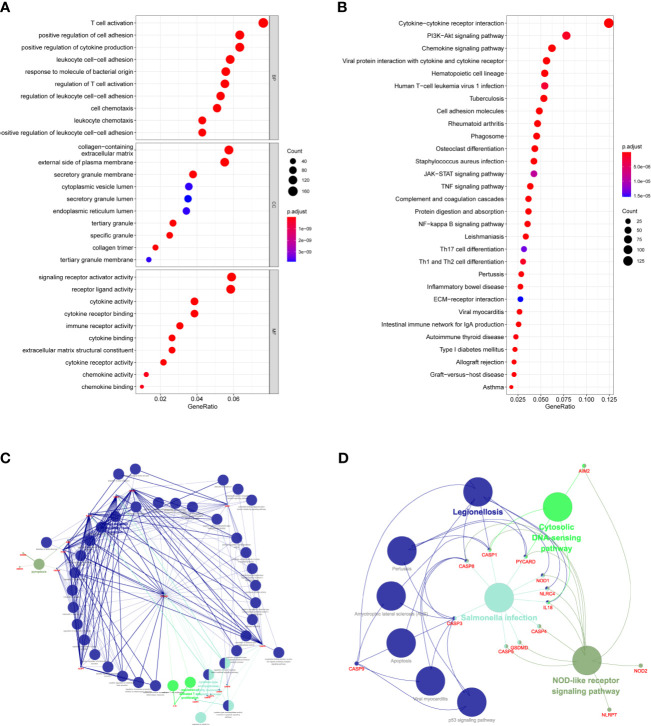
Functional analysis based on the DEGs between the two-risk groups in the TCGA cohort. **(A)** Analysis of GO enrichment for DEGs. **(B)** Analysis of KEGG enrichment for DEGs. **(C)** Result of ClueGO GO : BP enrichment. **(D)** Result of ClueGO KEGG pathway enrichment.

**Figure 8:**
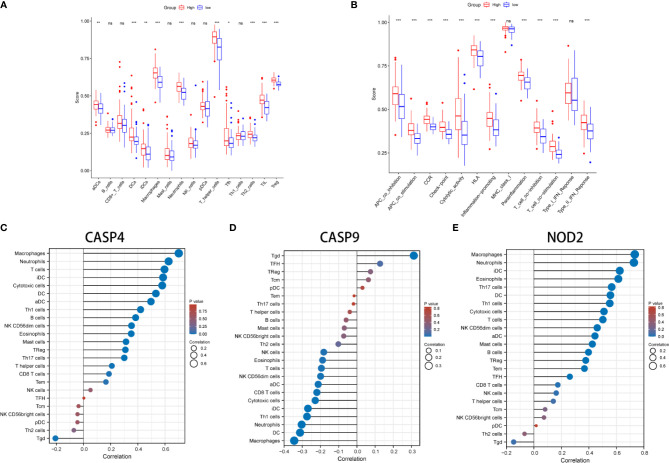
Comparison of the ssGSEA scores between different risk groups in the derivation cohort. The scores of 16 immune cells **(A)** and 13 immune-related functions **(B)** are displayed in boxplots. **(C–E)** The correlation between infiltrating immune cell types in GBM and three DEGs. *P < 0.05; **P < 0.01; ***P < 0.001; ns p > 0.05.

KEGG and GO function enrichment analyses were performed. GO was carried out in three functional ontologies: biological process (BP), cellular component (CC), and molecular function (MF). The DEGs were most enriched in biological processes, and specifically in immune-related processes such as T-cell activation, positive regulation of cell adhesion, positive regulation of cytokine production, and leukocyte cell−cell adhesion (*p* < 0.05; **Figure 7A**). In addition, KEGG pathway analyses indicated that the DEGs were highly enriched in cytokine-cytokine receptor interaction, PI3K−Akt signaling pathway, chemokine signaling pathway, hematopoietic cell lineage, rheumatoid arthritis, *Staphylococcus aureus* infection, etc. (*p* < 0.05; **Figure 7B**). Similarly, BP was performed using the ClueGO network of the KEGG pathways and GO (**Figures 7C, D**). To further explore the relationship between immune cell infiltrations and risk scores in the prognostic risk model, ssGSEA in R package GSVA was used to quantify the level of immune cell function or pathways in the cancer samples. Interestingly, the scores of most immune cell types (activated dendritic cells, macrophages, neutrophils, T helper cells, Th2 cells, tumor-infiltrating lymphocyte, and T regulatory cell) were significantly different between the two groups (**Figure 8A**; *p* < 0.05). Moreover, APC coinhibition, APC costimulation, cytokine-cytokine receptor, checkpoint, cytolytic activity, human leukocyte antigen, human leukocyte antigen, inflammation-promoting, parainflammation, T-cell coinhibition, T-cell costimulation, and type III FN response were significantly different between the two groups (**Figure 8B**; *p* < 0.05). This finding was consistent with results from GO and KEGG analysis. ssGSEA was used to analyze the correlation between the various infiltrating immune cell types in GBM and the expression levels of the three DEGs, and the results showed that they were closely related to infiltrating immune cells (**Figures 8C–E** and **Table S2**).

## Discussion

GBM is a highly invasive tumor, characterized by infiltration, aggressiveness, and resistance to treatment. The incidence of GBM is 3.2 per 100,000 population (20), and relapse has been reported in GBM patients treated with surgery alone. Despite the continuous improvements in surgical resection, chemotherapy, and radiation therapies, GBM remains difficult to treat and the overall mortality rates remain high (21). Thus, there is an urgent need to develop new prognostic models (22). Pyroptosis is a new lytic, and proinflammatory type of programmed cell death that is mainly involved in proinflammatory events (23). It is mediated by cysteine aspartic acid-specific protein kinases 1, 4, 5, and 11, which mainly depends on the poreforming activity of the Gasdermin protein family. At present, pyroptosis is reported to participate in the occurrence and development of various diseases, especially its dual role in promoting and inhibiting tumor formation and tumor microenvironment (24, 25). However, the prognostic value and mechanism of pyroptosis-related genes in GBM remain to be investigated. Thus, in this study, the expression of 33 pyroptosis-related genes in GBM tissues and their associations with OS was systematically explored. The results showed that 32 pyroptosis-related genes were differentially expressed between GBM and adjacent normal tissues in the derivation cohort. Moreover, a novel pyroptosis-related gene signature was constructed and validated and found to have good accuracy for predicting the survival of GBM patients. A total of three pyroptosis-related genes (*CASP4*, *CASP9*, and *NOD2*) were identified and included in the prognostic model.

Studies have demonstrated that dysregulation of *CASP4* is implicated in the initiation and development of many human diseases, including malignancies (26, 27). Abnormal expression of *CASP4* in clear cell renal cell carcinomas has been reported as an important prognostic factor affecting prognosis (27). *CASP9* is involved in many cellular processes, and its main role is an initiator caspase. Besides, it is an important therapy target for many diseases related to pyroptosis (28). A combination of *CASP9* overexpression with radiation has shown promise in enhancing the efficacy of tumor treatment (29). This is consistent with the current study findings. The role of *NOD2* in inducing innate and adaptive immunity is complicated and thought to play a decisive role in maintaining microbial tolerance at the intestinal barrier (30). More experiments and clinical studies are, however, needed to establish the role of *NOD2* in *GBM*.

The three pyroptosis-related genes were used to build a prognostic model with a good model fit, and high prediction power (0.921, 0.840, and 0.905 at 1, 3, and 5 years, respectively). The risk score of each patient was calculated using the prognostic model and was found to be an independent predictor of OS for GBM patients. These findings can contribute to the development of accurate and sensitive diagnostic and prognostic biomarkers for GBM. The three pyroptosis-related genes were further experimentally validated by qRT-PCR and Western blotting, thus providing strong evidence for the above conclusion.

The GO and KEGG enrichment analysis revealed significant enrichment of genes in immune-related pathways. In addition, the scores of most immune cell types (activated dendritic cell, macrophages, neutrophils, T helper cells, Th2 cells, tumor-infiltrating lymphocyte, and T regulatory cell) and APC coinhibition, APC costimulation, cytokine-cytokine receptor, checkpoint, cytolytic activity, human leukocyte antigen, human leukocyte antigen, inflammation-promoting, parainflammation, T-cell coinhibition, T-cell costimulation, and type III FN response were found to be statistically different between the two groups.

In summary, a novel prognostic model based on three pyroptosis-related genes was constructed and used to predict the prognosis of GBM patients. This model proved to be significantly associated with OS and can provide insights into the prediction of GBM prognosis.

## Data Availability Statement

The original contributions presented in the study are included in the article/**Supplementary Material**. Further inquiries can be directed to the corresponding authors.

## Ethics Statement

The studies involving human participants were reviewed and approved by Independent Ethics Committee of The First Affiliated Hospital of Zhengzhou University. The patients/participants provided their written informed consent to participate in this study.

## Author Contributions

X-TY, Xu-YL and L-XS designed experiments. L-YZ and Xi-YL carried out experiments and wrote the manuscript. X-TY, Xu-YL and L-XS performed manuscript review. All authors contributed to the article and approved the submitted version.

## Funding

This study received Fundamental research program funding of Ninth People's Hospital affiliated to Shanghai Jiao Tong University School of Medicine (No. JYZZ076), Clinical Research Program of Ninth People's Hospital, Shanghai Jiao Tong University School of Medicine (No. JYLJ201801, JYLJ201911), the China Postdoctoral Science Foundation (No. 2017M611585) and the National Natural Science Foundation of China (No. 81871458).

## Conflict of Interest

The authors declare that the research was conducted in the absence of any commercial or financial relationships that could be construed as a potential conflict of interest.

## Publisher’s Note

All claims expressed in this article are solely those of the authors and do not necessarily represent those of their affiliated organizations, or those of the publisher, the editors and the reviewers. Any product that may be evaluated in this article, or claim that may be made by its manufacturer, is not guaranteed or endorsed by the publisher.

## Supplementary Material

Supplementary Figure 1 Processes of LASSO Cox model fitting. **(A)** The profile of coefficients in the model at varying levels of penalization plotted against the log (lambda) sequence. **(B)** Tenfold crossvalidated error (first vertical line equals the minimum error, whereas the second vertical line shows the crossvalidated error within 1 standard error of the minimum).

Supplementary Table 1 Thirty-three pyroptosis-related genes.

Supplementary Table 2 The correlation between infiltrating immune cell types in GBM and three DEGs.

## Abbreviations

TCGA, The Cancer Genome Atlas; DEGs, differentially expressed genes; OS, overall survival; ROC, receiver operating characteristic; FDR, false-discovery rate; GO, Gene Ontology; KEGG, Kyoto Encyclopedia of Genes and Genomes; ssGSEA, single-sample gene set enrichment analysis; PCA, principal component analysis; AUC, area under the curve; HR, hazard ratio; CI, confidence interval; APC, antigen presenting cell; aDC, activated dendritic cell; iDC, immature dendritic cell; pDC, plasmacytoid dendritic cell; Tcm, T central memory; Tfh, T follicular helper cell; TIL, tumor-infiltrating lymphocyte; Tgd, T gamma delta; HLA, human leukocyte antigen; CCR, cytokine-cytokine receptor.
