# Supplementary figures and images for: A Pyroptosis-Related Gene Signature for Predicting Survival in Glioblastoma
Source: Front Oncol. 2021 Aug 17;11:697198. doi: 10.3389/fonc.2021.697198 (PMC8416108; doi:10.3389/fonc.2021.697198)

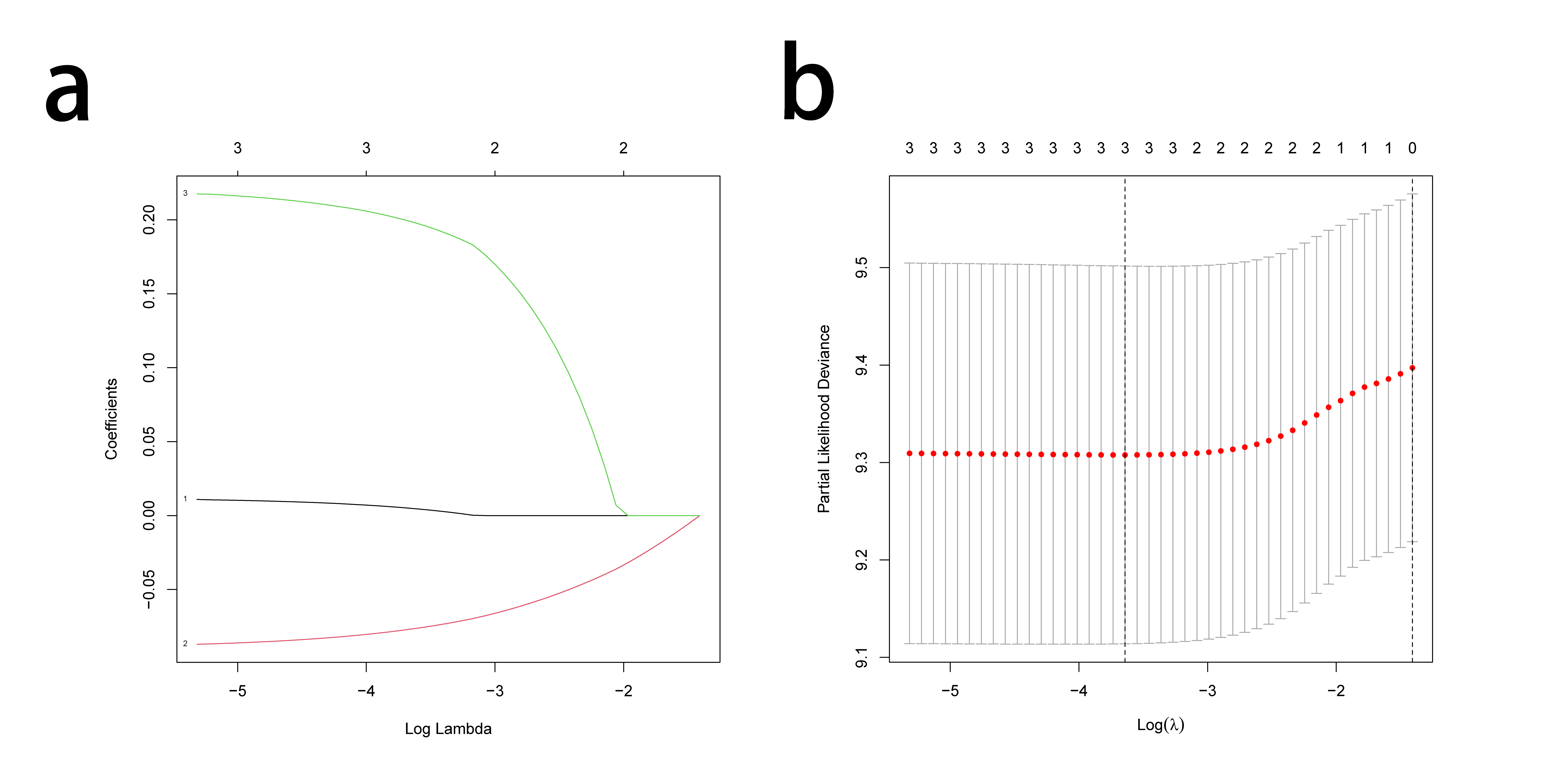

Supplement: Supplementary Figure 1 — Processes of LASSO Cox model fitting. (A) The profile of coefficients in the model at varying levels of penalization plotted against the log (lambda) sequence. (B) Tenfold crossvalidated error (first vertical line equals the minimum error, whereas the second vertical line shows the crossvalidated error within 1 standard error of the minimum). [file Image_1.tif]
