# Supplementary material for: A Pyroptosis-Related Gene Signature for Predicting Survival in Glioblastoma
Source: Front Oncol. 2021 Aug 17;11:697198. doi: 10.3389/fonc.2021.697198 (PMC8416108; doi:10.3389/fonc.2021.697198)
Supplement: Supplementary Table 1 — Thirty-three pyroptosis-related genes. [file Table_1.docx]

Table S1: 33 pyroptosis-related genes

AIM2

CASP1

CASP3

CASP4

CASP5

CASP6

CASP8

CASP9

ELANE

GPX4

GSDMA

GSDMB

GSDMC

GSDMD

GSDME

IL18

IL1B

IL6

NLRC4

NLRP1

NLRP2

NLRP3

NLRP6

NLRP7

NOD1

NOD2

PJVK

PLCG1

PRKACA

PYCARD

SCAF11

TIRAP

TNF
